# More than sleep problems? Testing five key health behaviors as reasons for quality of life issues among shift workers

**DOI:** 10.1186/s12955-024-02269-4

**Published:** 2024-07-03

**Authors:** Yuxin Chen, Kaiyi Deng, Ian M. Hughes, Claire E. Smith, Hongdao Meng, Minh Quan Le, Min Sun, Xianyan Zhang, Danping Liu

**Affiliations:** 1 West China School of Public Health and West China Fourth Hospital, Sichuan University, Chengdu, Sichuan China; 2 Jockey Club School of Public Health and Primary Care, The Chinese University of Hong Kong, Hong Kong SAR, China; 3 Department of Psychology, North Carolina State University, Raleigh, North Carolina USA; 4 School of Aging Studies, University of South Florida, Tampa, Florida USA

**Keywords:** Shift work, Quality of life, Health behaviors

## Abstract

**Background:**

The shift work schedule is a common work arrangement that can disrupt typical sleep-wake rhythms and lead to negative health consequences. The present study aims to examine the effect of shift work on health-related quality of life (QoL) and explore potential behaviorial mediators (i.e., sleep, eating, exercise, smoking, drinking).

**Methods:**

A cross-sectional survey was conducted among 4,449 petroleum workers in southwest China. Data on shift work status, health behaviors, and physical and mental health QoL were collected. We tested our model using path analysis and the Monte Carlo approach among 2,129 included participants.

**Results:**

After adjusting for covariates, shift work did not exhibit a significant direct association with QoL. However, shift work indirectly related to poorer physical health quality of life via less frequent healthy food consumption; shift work also indirectly related to poorer mental health QoL via both less frequent healthy food consumption and physical exercise. No significant indirect effects were found via sleeping, smoking, or drinking.

**Conclusions:**

Results suggest that shift work presents a challenge for QoL among Chinese petroleum workers due to their lesser engagement in two specific health behaviors: healthy eating and physical exercise. Healthy eating and exercise may present an even more prominent threat to shift workers’ QoL than sleep and substance use. Strategies targeting shift work schedule as well as eating and exercise behaviors may help protect against poor QoL and adverse physical and mental health outcomes in this vulnerable group.

**Supplementary Information:**

The online version contains supplementary material available at 10.1186/s12955-024-02269-4.

## Introduction

In today’s global and industrialized society, a substantial and growing proportion of the workforce operates on non-standard work schedules [1, 2], outside of the standard day shift (i.e., between 8:00 A.M. to 6:00 P.M, Monday to Friday). The shift work, so to speak, has been especially prominent within industries that maintain 24-hour operations, such as the petroleum industry [3]. Unfortunately, by altering sleep/wake cycles to accommodate shift work schedules, shift work disrupts the internal, biological clock (i.e., circadian rhythm), which impedes shift workers’ ability to live a normal and healthy life outside of work [4]. Shift work is therefore a significant risk factor for a variety of serious health conditions including obesity, cardiovascular disease, and cancers as well as lower quality of life (QoL) [5–9]. Now, empirical work is needed to uncover the major threats, or constellation of threats, to QoL in shift workers, as a high-risk and growing occupational group.

**Fig 1:**
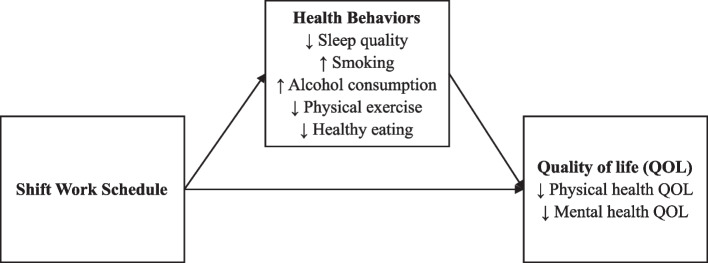
Hypothesized model

We apply an integrated theoretical framework, across QoL, ecological systems, and health behavior theory, to test key health behaviors (i.e., poor sleep, eating, and physical exercise and excessive smoking and drinking) as primary reasons for QoL issues among shift workers. QoL encompasses both a subjective sense of well-being and objective indicators, like health status [10]. Modern definitions consider both mental and physical health as central to overall health and QoL [11–13]. According to conceptual models of QoL, people help determine their own QoL through their actions, but their actions are guided by their broader environments [14]. More specifically, our environments shape our functional status (i.e., ability to perform physically and psychologically within one’s life roles) which, in turn, influences overall assessments of QoL. Work – including one’s industry, organization, and particular job – is a micro-environmental system that powerfully impacts individual behavior and well-being [15, 16]. In line with this model, we position shift work as an influential aspect of one’s work that may shape functional status, indicated by health behaviors, ultimately leading to differences in physical and mental health QoL. The hypothesized model is illustrated through MSOffice (see Fig 1).

Due to the discussed disruptions to circadian rhythm caused by shift schedules, most previous research focuses on sleep as the primary health behavior impaired by shift work [17, 18]. Indeed, poor sleep quality is common complaints among shift workers, including in the oil rig industry [19]. In fact, sleep problems are so prevalent in this group that the term *shift-work disorders* was developed, characterized by excessive sleepiness and insomnia symptoms according to the American Academy of Sleep Medicine [20]. Yet researchers increasingly urge that health behaviors tend to co-occur, such that people who engage in one healthy behavior, like good sleep, are also more likely to engage in other healthy behaviors (e.g., frequent physical exercise, low alcohol consumption) [21, 22]. Further, on the other side of the spectrum, multiple unhealthy behaviors present heightened risk compared to one alone [23–25]. Within the shift work literature, “no single mechanism seems to be working” (p.96) when considering the consequences of shift work via health behaviors [26]. For these reasons, it may be more accurate and informative to consider a variety of key health behaviors to determine which single or multiple behavior(s) present the strongest threat to QoL in shift workers. In addition to sleep, we focus on four health behaviors that have, on their own, been empirically identified as heightened concerns for shift workers: physical exercise, healthy eating, cigarette smoking, and alcohol consumption [27–31]. In total, our model brings a theoreticaly grounded explanation to healthy behavior and QoL research – a literature that is often criticized for leaning conceptual and atheoretical [32]. Moreover, because health behaviors represent a modifiable risk factor for health, disease, and death, results will point to specific, intervenable behavior(s) that present a promising opportunity to increase shift workers’ QoL [33].

## Method

### Participants and procedure

Using cluster sampling method, all the participants were employees in a petroleum company located in southwest China. This population was chosen as a large group of employees that are part of a booming industry but face severe threats to their health and quality of life, due to occupational stressors including high risk for fatal injury, adverse physical conditions, and isolation from friends and family (Chen et al., 2003). The purpose, the significance, and the content of the survey were introduced to the participants by the health management department of Southwest Petroleum Company. And the online questionnaire was distributed and filled with the informed consent of the workers. A total of 4,449 participants completed our survey materials. A unique code, important for establishing confidentiality, was used to match each respondent.

According to the study objective, we excluded participants who work off-site, and those whose shift work situation was ‘others’, as we considered they were not our target group. Also, individuals with incomplete sleep quality scores or life quality scores were excluded. Finally, our study sample included 2129 on-site petroleum workers. Employees in this sample were primarily male (97%), married (74%), and were an average of 36.84 years of age (*SD* = 8.69). Most of our sample worked in multiple shifts as opposed to a fixed day shift (62%). See Table 1 for detailed sample information.

**Table 1:** Sample characteristics

Variable	N (%)
**Gender**	
Men	2074 (97.4)
Women	55 (2.6)
**Age (years old)**	
29 or lower	561 (26.4)
30-39	838 (39.4)
40-49	585 (27.5)
50+	145 (6.8)
**Marital status**	
Unmarried (never married or divorced)	554 (26.1)
Married	1575 (74.0)
**Education**	
High school or lower	1885 (88.5)
Undergraduate degree	228 (10.7)
Master’s degree and above	16 (0.8)
**Income (in Yuan, per month)**	
0-4,999	508 (23.9)
5,000-9,999	1461 (68.6)
10,000+	154 (7.2)
**Job tenure (in years)**	
0-9	815 (38.3)
10-19	1053 (49.5)
20+	259 (12.2)
**Job level**	
None	802 (37.7)
Junior	512 (24.0)
Intermediate	329 (15.5)
Senior	486 (22.8)
**Shift work**	
Fixed day shift	812 (38.1)
Shift work	1317 (61.9)

*N* = 2,129

### Measures

#### Shift work

Shift work was assessed using a single item that read “What is your shift situation?”. Participants selected from one of the following options: “Fixed day shift”, “Two shifts”, “Three shifts”, or “Other”. Participants who selected “other” were omitted. We otherwise split our sample into two groups: one that worked a typical, fixed day shift, and one that worked multiple shifts.

#### Health behaviors

Poor *sleep quality* was assessed using a composite sleep quality score across seven dimensions (i.e., sleep duration, sleep disturbance, sleep onset latency, daytime dysfunction, habitual sleep efficiency, subjective sleep quality, and use of sleeping medication) from the Pittsburgh Sleep Quality Index (PSQI) [34]. Participants responded to 19 items assessing their sleep over the past one month before they fulfill the questionnaire. Each of the seven components is scored from 0 to 3 and yielding a global score from 0 to 21, with higher scores indicating worse sleep. Great test-retest reliability and validity for identifying cases with sleep disturbances were reported [35]. In the current sample, Cronbach α =0.800. Smoking behavior was operationalized as participants’ self-reported average number of daily cigarettes smoked over the last month (i.e., “In the last month, you have smoked an average of cigarettes every day.”). Alcohol consumption was assessed using a single item assessing frequency of drinking (i.e., “Do you drink alcohol?” on a scale from 0 = No to 4 = Drink almost every day)). Weekly physical exercise frequency was assessed using two items: (1) “In the past six months, have you regularly participated in physical exercise or outdoor activities in your spare time?” (1 = yes, 5 = no) and (2) “How many times a week do you participate in physical exercise or outdoor activities on average?” (1 = <1 time per week, 2 = 1-2 times per week, 3 = 3-4 times per week, 4 = >5 times per week). Participants who reported never engaging in physical exercise were assigned a score of 0 for exercise frequency. Finally, the consumption of healthy foods was operationalized as the average number of fruits and vegetables (both scaled 1 = <1 day per week, 5 = Eat daily) eaten in one’s daily diet. Frequent consumption of fruits and vegetables are widely recommended as part of a healthy diet and is connected to lower risk of a variety of health conditions, making it a key aspect of healthy eating [36–39].

#### Quality of life

Mental and physical health were assessed using the Chinese-translated Short Form-12 Health Survey, version 2 (SF-12 v2), which was the short form health survey directly from the SF-36 v2 [40]. ]). Two summary measures, physical component summary (PCS) and mental component summary (MCS), were derived from the 12 items and scored from 0 to 100 according to the scoring manual [41, 42]. The SF-12 v2 includes 12 items that yielded 8 scale scores aggregated as the PCS (physical functioning [PF], role-physical [RP], bodily pain [BP], general health [GH]) and MCS (vitality [VT], social functioning [SF], role-emotional [RE], and mental health [MH]). The criterion validity and reliability of SF-12 v2 were assessed and proved to be reliable. In the current sample, Cronbach α =0.683.

## Results

### Preliminary results

Descriptive statistics for and correlations between our focal variables can be found in Table 2. Before testing our hypotheses, we sought to determine the discriminant validity of our variables as measured using confirmatory factor analysis. First, we tested the fit of our eight-factor measurement model, consisting of our independent variable (shift work), mediators (poor sleep quality, smoking behavior, drinking behavior, weekly exercise frequency, and consumption of healthy food), and dependent variables (mental and physical health QoL). Model parameters were estimated using diagonally weighted least squares estimation, which is optimal when dealing with non-normal or ordinal data [43, 44]. The measurement model demonstrated satisfactory fit: χ^2^(13) = 27.48, *p* < .05; CFI: .99; RMSEA: .02; SRMR: .02 [45]. To assess potential influence of common method bias (CMB) on our cross-sectional measurement, we then compared the intended eight-factor measurement model to a single-factor model which loaded all variables onto one latent (i.e., common method) factor. Model fit statistics better than or comparable to our intended model would indicate that CMB may be a substantial threat to the accuracy of results [46]. However, the single-factor model demonstrated significantly worse fit than the measurement model (χ^2^(35) = 324.73, *p* < .01; CFI: .86; RMSEA: .06; SRMR: .07): Δχ^2^(22) = 297.25, *p* < .01, reducing concerns about CMB. Thus, we proceeded with hypothesis testing.

**Table 2:** Descriptive statistics and intercorrelations of key variables

Variable	*M*	*SD*	1	2	3	4	5	6	7	8	9	10
1. Gender (0=man, 1=woman)	1.03	0.16										
2. Age	36.84	8.69	.01									
3. Marital status (0=unmarried, 1=married)	1.86	0.49	.03	.49**								
4. Shift (0=day, 1=multiple)	1.62	0.49	-.15**	-.17**	-.05*							
5. Poor sleep quality	5.98	3.56	.01	.03	.04	.07**						
6. Smoking frequency	7.41	8.28	-.14**	.07**	.08**	.03	.12**					
7. Alcohol consumption	2.00	1.00	-.15**	.06**	.04	-.06**	.09**	.20**				
8. Physical exercise	1.04	1.32	.04*	.21**	.05*	-.14**	-.18**	-.09**	-.05*			
9. Healthy eating	3.17	1.19	.10**	.01	-.02	-.11**	-.16**	-.11**	-.11**	.19**		
10. Physical health QOL	84.68	25.71	.05*	.00	.01	-.05*	-.28**	-.06**	-.03	.09**	.17**	
11. Mental health QOL	65.66	20.99	.02	.11**	.03	-.10**	-.42**	-.06**	-.06**	.18**	.20**	.36**

*SD* Standard deviation, *Smoking frequency* Number of cigarettes smoked per day, *Alcohol consumption* Frequency of drinking alcohol, *Physical exercise* Times per week a person engages in physical exercise activities, *Healthy eating* average number of fruits and vegetables eaten in one’s daily diet, *QOL* Quality of life

*N* = 2,129. * indicates *p* < .05. ** indicates *p* < .01. *M* = Mean

### Hypothesis testing

To test our hypotheses, we conducted path analysis using the lavaan package in R [47]. When configuring our path models, we controlled for gender, age, and marital status, due to their previously established connections to health behaviors and mental and physical health outcomes [48–54]. We allowed our mediators to covary with one another in line with best practice recommendations, as health behaviors are theoretically and empirically intertwined [21, 22, 55]. When generating confidence intervals for our indirect effects, we relied on the Monte Carlo approach with 20,000 replications [56]; this method is often used for better interpreting the significance of mediating effects in path models. We configured two path models: Model 1, focused on physical health QoL as a dependent variable, and Model 2, focused on mental health QoL as a dependent variable.

#### Physical health quality of life

Beginning with Model 1 (see Table 3; χ^2^(2) = 57.99, CFI = .94, RMSEA = .11, SRMR = .02), shift work was not directly related to physical health QOL (β = -.01, *p* = .75). Regarding direct effects, shift work shared significant direct effects with drinking alcohol (β = -.10, *p* < .01), physical exercise (β = -.12, *p* < .01), and consumption of healthy foods (β = -.11, *p* < .01) but not smoking (β = .01, *p* = .59) or sleep (β = .04, *p* = .09). Of note, shift work unexpectedly related to less frequent alcohol consumption, rather than more. When modeling direct effects from our mediators to dependent variable, only poor sleep quality (β = -.26, *p* < .01) and consumption of healthy foods (β = .13, *p* < .01) were significantly related to physical health QOL. One significant indirect effect was observed, namely shift work to physical health QOL through consumption of healthy foods (β = -.01, 95%CI[-.023, -.006], *p* < .01; variance accounted for: 78.9%). Put differently, shift work is negatively related to physical health QOL through less frequent consumption of healthy foods.^1^

**Table 3:** Direct and indirect effects for model 1

**Paths**	**Est.**	***SE***	**95% CI-L**	**95% CI-U**
Shift – Sleep Quality	0.04	0.03	-0.01	0.09
Shift – Smoking	0.01	0.03	-0.04	0.06
Shift – Drinking	-0.10^**^	0.03	-0.15	-0.05
Shift – Exercising	-0.12^**^	0.03	-0.17	-0.07
Shift – Healthy Eating	-0.11^**^	0.02	-0.16	-0.06
Shift – Physical Health QOL	-0.01	0.03	-0.06	0.04
Sleep – Physical Health QOL	-0.26^**^	0.03	-0.32	-0.20
Smoking – Physical Health QOL	-0.01	0.03	-0.06	0.04
Drinking – Physical Health QOL	0.02	0.03	-0.03	0.07
Exercising – Physical Health QOL	0.04	0.03	-0.01	0.09
Healthy Eating – Physical Health QOL	0.13^**^	0.03	0.08	0.18
Shift – Sleep Quality – Physical Health QOL	-0.01	0.01	-0.02	0.00
Shift – Smoking – Physical Health QOL	0.00	0.00	-0.00	0.00
Shift – Drinking – Physical Health QOL	-0.00	0.00	-0.01	0.00
Shift – Exercising – Physical Health QOL	-0.01	0.00	-0.01	0.00
Shift – Healthy Eating – Physical Health QOL	-0.01^**^	0.00	-0.02	-0.01

*95% CI-L* Lower bound 95% confidence interval, *95% CI-U* Upper bound 95% confidence interval, *QOL* Quality of Life

*N* = 2,129. Est. indicates standardized path estimate. *SE* indicates the standard error of the respective path estimate

^*^ = *p* < .05, ** = *p* < .01

#### Mental health quality of life

Moving on to Model 2 (see Table 4; χ^2^(2) = 57.37, CFI = .95, RMSEA = .11, SRMR = .02), shift work was not directly related to mental health QOL (β = -.03, *p* = .30). Direct effects from shift work to our mediators mirrored those found in Model 1 (i.e., significant effects for less drinking [β = -.10, *p* < .01], less physical exercise [β = -.12, *p* < .01], and less consumption of healthy foods [β = -.11, *p* < .01]). When modeling direct effects from our mediators to mental health QOL, poor sleep quality (β = -.39, *p* < .01), physical exercise frequency (β = .08, *p* < .01), and consumption of healthy foods (β = .13, *p* < .01) were significantly related to mental health QOL, but not drinking (β = -.02, *p* = .58) or smoking (β = .00, *p* = .99). Multiple significant indirect effects were observed, namely shift work to mental health QOL through weekly exercise frequency (β = -.01, 95%CI[-.018, -.003], *p* < .05; variance accounted for: 15.8%) and consumption of healthy foods (β = -.01, 95%CI[-.023, -.007], *p* < .01, variance accounted for: 22.2%). Put differently, shift work is negatively related to mental health QOL through a decrease in weekly exercise frequency and consumption of healthy foods.

**Table 4:** Direct and indirect effects for model 2

**Paths**	**Est.**	***SE***	**95% CI-L**	**95% CI-U**
Shift – Sleep Quality	0.04	0.03	-0.01	0.09
Shift – Smoking	0.01	0.03	-0.04	0.06
Shift – Drinking	-0.10^**^	0.03	-0.15	-0.05
Shift – Exercising	-0.12^**^	0.03	-0.17	-0.07
Shift – Healthy Eating	-0.11^**^	0.02	-0.16	-0.06
Shift – Mental Health QOL	-0.03	0.03	-0.08	0.03
Sleep – Mental Health QOL	-0.39^**^	0.03	-0.44	-0.33
Smoking – Mental Health QOL	0.00	0.03	-0.05	0.05
Drinking – Mental Health QOL	-0.02	0.03	-0.07	0.04
Exercising – Mental Health QOL	0.08^**^	0.03	0.03	0.14
Healthy Eating – Mental Health QOL	0.13^**^	0.03	0.08	0.18
Shift – Sleep Quality – Mental Health QOL	-0.02	0.01	-0.04	0.00
Shift – Smoking – Mental Health QOL	0.00	0.00	-0.00	0.00
Shift – Drinking – Mental Health QOL	0.00	0.00	-0.00	0.01
Shift – Exercising – Mental Health QOL	-0.01^*^	0.00	-0.02	-0.00
Shift – Healthy Eating – Mental Health QOL	-0.01^**^	0.00	-0.02	-0.01

*95% CI-L* Lower bound 95% confidence interval, *95% CI-U* Upper bound 95% confidence interval, *QOL* Quality of Life

*N* = 2,129. Est. indicates standardized path estimate. *SE* indicates the standard error of the respective path estimate

^*^ = *p* < .05, ** = *p* < .01

## Discussion

Shift work arrangements are not only increasingly prevalent [1, 2] but present a serious threat to workers’ health and QoL. Yet the specific reasons for QoL challenges among this growing group and, as a result, promising intervention points are missing in the current literature. Applying an integrated theoretical framework, our study samples a large group of petroleum workers, half of whom worked on a shift schedule, to assess *whether* and *which* health behaviors link shift work schedules to poorer QoL. Interestingly, shift work in and of itself did not directly relate to poorer physical or mental health QoL. Instead, worse health behaviors, specifically lesser consumption of healthy food and lesser physical exercise engagement, fully mediated the association between shift work schedules and QoL issues.

Although past research has primarily focused on sleep as a health behavior that is difficult for shift workers to achieve [17, 18], eating and exercising emerged as more influential for their QoL when a broader constellation of key health behaviors were considered simultaneously. Past research points to several potential explanations for this finding. Despite existing evidence that shift work hinders healthy sleep, sleep health is increasingly acknowledged as a substantial challenge for all workers, who report concerningly prevalence of short sleep and sleep disorder symptoms [57]. Thus, poor sleep and related consequences may be common across both shift and non-shift workers in our sample, especially given the high-stress occupational context [58]. Further, evidence suggests that some shift workers may be able to adapt their sleep within shift work schedules [59, 60], whereas healthy eating and exercise options (e.g., certain grocery stores, restaurants, indoor and outdoor exercise facilities, and fitness classes) may be limited during non-standard times regardless of personal adaptation to a shift work schedule. As such, although sleep is a notable issue for shift workers, its influence may not be stronger above and beyond other key health behaviors (i.e., eating and exercise) when such behaviors are considered simultaneously, in line with best practice recommendations [23–25].

Like sleep, neither smoking nor alcohol consumption emerged as significant mediators in the present study. In fact, shift work did not significantly relate to smoking frequency at all and unexpectedly negatively related to alcohol consumption, indicating that shift workers tend to drink *less* than those on standard day schedules. Cultural context may be relevant to the smoking results. Much of the published research focuses on largely white, Western samples, where, in the United States for example, the percentage of cigarette smokers is less than half of China [61, 62]. All workers in the present sample may be more likely to smoke than the global average due to the national context, regardless of work schedule. Although shift workers are also typically positioned as more likely to drink alcohol, null and negative associations between shift work and alcohol consumption have been found previously [30]. One possible reason for the unexpected negative association found here is that shift workers in the demanding petroleum industry simply have less time and opportunity to consume alcohol. Another explanation is that shift workers may choose to drink less alcohol because doing so would compound the existing challenge they have remaining attentive and focused at work due to their shift work schedule [63].

Overall, we find that shift workers may struggle to maintain sufficient QoL because these atypical work schedules impede their ability and/or motivation to eat healthfully and engage in regular physical activity. This finding provides several novel insights into the research literature and provide practical suggestions for organizations and employees involved in shift work arrangements. First, this research extends beyond previous findings *that* shift work presents a threat to employees’ health and QoL to also explain *why* these arrangements may be detrimental. Focusing on explanatory variables (i.e., health behaviors) is essential to developing strong theory; under-examination of explanatory mediators may be a key reason previous research in this area has been characterized as atheortical [27, 64]. In line with existing theoretical models on QoL and health behavior, shift work status seems to function as an important element of a person’s work environment, guiding individual engagement in interrelated health behaviors (i.e., eating, exercise) outside of work that play a part in determining QoL [14, 21, 22]. Second, our results suggest that healthy eating and regular physical exercise may be more urgent points of intervention for supporting QoL among shift workers than previously thought. Existing research has recently begun to develop related interventions in shift workers, including those that target multiple relevant health behaviors, but scholars urge that further work is needed to refine these interventions and validate them in high-risk occupations such as the petroleum industry [65–67]. We hope that our findings directly motivate targeted, multi-facted health behavior intervention development and testing among high-risk shift workers to protect their vulnerable QoL.

## Limitations and future directions

There are limitations with our research that are important to consider. Primarily, the data were monomethod and cross-sectional, meaning it was collected via self-report method only at a single point in time). As mentioned, these data characteristics increase the risk of distorted results via common method bias or CMB [46]. That said, we proactively tested the potential influence of CMB in our data and found that our intended measurement model demonstrated better fit to our data than did the latent method model, indicating that our results are not likely to be primarily the result of CMB. In addition, though, cross-sectional data are typically not optimal for tests of indirect effects because they cannot assess change over time [68]. Designs such as ours are, however, suitable for initial explorations of phenomena [69]. These results therefore set a foundation for scholars to build upon but cannot explicitly test the hypothesized causal chain of events, despite their grounding in theory and past research. As such, future research should continue to investigate the joint mediating effects of health behaviors on the relations between shift work and QoL using longitudinal, within-person designs [70]. Scholars should also collect other or objective reports of key variables to combat CMB measurement concerns. For example, researchers could use actigraphy to assess sleep quality and quantity and heartrate or pedometer data to assess physical exercise. Additionally, other indicators of QoL should be assessed in the future to examine the robustness of the result in our research. Finally, our sample of Chinese petroleum workers represent a vulnerable group but that certainly may not be generalizable to a general worker or even general shift worker population, given their exposure to extreme stress and even threats to basic safety. The oil industry employs millions, making this population a large and valuable one to study, but additional research is needed to determine whether our findings transfer to other shiftwork populations.

## Conclusion

The goal of the present research was to explore the main reasons that shift workers experience heightened vulnerability to poor QoL. We positioned five key health behaviors as potential mediators linking shift work and QOL. Among a large sample of Chinese petroleum workers, we found that shift work and physical health QOL are related through less frequent healthy food consumption, whereas shift work and mental health QOL are related through less frequent healthy food consumption and physical exercise. Healthy eating and physical exercise may present heightened challenges for QOL among shift workers, even beyond traditionally emphasized health behaviors in this group such as sleep and substance use.

### Supplementary Information

Supplementary Material 1. 

## Data Availability

The datasets used and/or analysed during the current study are available from the corresponding author on reasonable request.

## Footnotes

1 As requested by the review team, we have included alternative structural model tests (i.e., a model that combined mental and physical health QOL into one outcome and a model that included both mental and physical health QOL simultaneously) in our Supplementary Material.
