# Supplementary material for: More than sleep problems? Testing five key health behaviors as reasons for quality of life issues among shift workers
Source: Health Qual Life Outcomes. 2024 Jul 3;22:52. doi: 10.1186/s12955-024-02269-4 (PMC11220953; doi:10.1186/s12955-024-02269-4)
Supplement: Supplementary file 1 — Supplementary Material 1. [file 12955_2024_2269_MOESM1_ESM.docx]

**Supplementary Material**

**Sensitivity Analyses**

Below, we report—as requested by the review team—supplementary analyses that consider alternative models.

1. Model results collapsing mental and physical health quality of life into one outcome (overall QOL):

Direct Effects

Shift work ~ Sleep: β = .04, *p* = .08

Shift work ~ Smoking: β = .01, *p* = .59

Shift work ~ Drinking: β = -.10, *p* < .01

Shift work ~ Exercise: β = -.12, *p* < .01

Shift work ~ Consumption of healthy foods: β = -.11, *p* < .01.

Sleep ~ Overall QOL: β = -.38, *p* < .01

Smoking ~ Overall QOL: β = -.01, *p* = .78

Drinking ~ Overall QOL: β = .00, *p* = .89

Exercise ~ Overall QOL: β = .07, *p* = < .01

Consumption of healthy foods: β = .16, *p* < .01

Indirect Effects

Shift work ~ Sleep ~ Overall QOL: β = -.02, *p* = .09

Shift work ~ Smoking ~ Overall QOL: β = .00, *p* = .81

Shift work ~ Drinking ~ Overall QOL: β = .00, *p* = .89

Shift work ~ Exercise ~ Overall QOL: β = -.01, *p* < .05

MCCIs: [-.02, -.00]

Shift work ~ Consumption of healthy foods ~ Overall QOL: β = -.02, *p* < .01

MCCIs: [-.03, -.01]

1. Model including both mental and physical health quality of life into the same model:

Direct Effects

Shift work ~ Sleep: β = .04, *p* = .08

Shift work ~ Smoking: β = .01, *p* =.59

Shift work ~ Drinking: β = -.10, *p* < .01

Shift work ~ Exercise: β = -.12, *p* < .01

Shift work ~ Consumption of healthy foods: β = -.11, *p* < .01

Sleep ~ Mental health QOL: β = -.39, *p* < .01

Smoking ~ Mental health QOL: β = .00, *p* = .99

Drinking ~ Mental health QOL: β = -.02, *p* = .58

Exercise ~ Mental health QOL: β = .08, *p* < .01

Consumption of healthy foods ~ Mental health QOL: β = .13, *p* < .01

Sleep ~ Physical health QOL: β = -.26, *p* < .01

Smoking ~ Physical health QOL: β = -.01, *p* = .68

Drinking ~ Physical health QOL: β = .02, *p* = .48

Exercise ~ Physical health QOL: β = .04, *p* = .13

Consumption of healthy foods ~ Physical health QOL: β = .13, *p* < .01

Indirect Effects

Shift work ~ Sleep ~ Mental health QOL: β = -.02, *p* = .09

Shift work ~ Smoking ~ Mental health QOL: β = .00, *p* = .99

Shift work ~ Drinking ~ Mental health QOL: β = .00, *p* = .59

Shift work ~ Exercise ~ Mental health QOL: β = -.01, *p* < .05

MCCIs: [-.02, -.00]

Shift work ~ Consumption of healthy foods ~ Mental health QOL: β = -.01, *p* < .01

MCCIs: [-.02, -.01]

Shift work ~ Sleep ~ Physical health QOL: β = -.01, *p* = .10

Shift work ~ Smoking ~ Physical health QOL: β = .00, *p* = .74

Shift work ~ Drinking ~ Physical health QOL: β = -.00, *p* = .49

Shift work ~ Exercise ~ Physical health QOL: β = -.01, *p* = .15

Shift work ~ Consumption of healthy foods ~ Mental health QOL: β = -.01, *p* < .01

MCCIs: [-.02, -.01]
